# Sorting nexin 12 interacts with BACE1 and regulates BACE1-mediated APP processing

**DOI:** 10.1186/1750-1326-7-30

**Published:** 2012-06-18

**Authors:** Yonghao Zhao, Yunshu Wang, Jiaye Yang, Xin Wang, Yingjun Zhao, Xian Zhang, Yun-wu Zhang

**Affiliations:** 1 Fujian Provincial Key Laboratory of Neurodegenerative Disease and Aging Research, College of Medicine, Xiamen University, Xiamen, Fujian, 361005, People’s Republic of China; 2 School of Pharmaceutical Sciences, Xiamen University, Xiamen, Fujian, 361005, People’s Republic of China; 3 Neurodegenerative Disease Research Program, Sanford-Burnham Medical Research Institute, La Jolla, CA, 92037, USA

**Keywords:** β-amyloid, β-amyloid precursor protein, β-site APP cleaving enzyme 1, Alzheimer’s disease, Intracellular trafficking, Sorting nexin 12.

## Abstract

**Background:**

β-site APP cleaving enzyme 1 (BACE1) cleaves β-amyloid precursor protein (APP) to initiate the production of β-amyloid (Aβ), the prime culprit in Alzheimer’s disease (AD). Dysregulation of the intracellular trafficking of BACE1 may affect Aβ generation, contributing to AD pathology. In this study, we investigated whether BACE1 trafficking and BACE1-mediated APP processing/Aβ generation are affected by sorting nexin 12 (SNX12), a member of the sorting nexin (SNX) family that is involved in protein trafficking regulation.

**Results:**

Herein, we find that SNX12 is widely expressed in brain tissues and is mainly localized in the early endosomes. Overexpression of SNX12 does not affect the steady-state levels of APP, BACE1 or γ-secretase components, but dramatically reduces the levels of Aβ, soluble APPβ and APP β-carboxyl terminal fragments. Downregulation of SNX12 has the opposite effects. Modulation of SNX12 levels does not affect γ-secretase activity or *in vitro* β-secretase activity. Further studies reveal that SNX12 interacts with BACE1 and downregulation of SNX12 accelerates BACE1 endocytosis and decreases steady-state level of cell surface BACE1. Finally, we find that the SNX12 protein level is dramatically decreased in the brain of AD patients as compared to that of controls.

**Conclusion:**

This study demonstrates that SNX12 can regulate the endocytosis of BACE1 through their interaction, thereby affecting β-processing of APP for Aβ production. The reduced level of SNX12 in AD brains suggests that an alteration of SNX12 may contribute to AD pathology. Therefore, inhibition of BACE1-mediated β-processing of APP by regulating SNX12 might serve as an alternative strategy in developing an AD intervention.

## Background

A major pathological hallmark of Alzheimer’s disease (AD) is the formation of senile plaques in the brain. The major components of senile plaques are heterogeneous small peptides called β-amyloid (Aβ) [1]. Aβ peptides are derived from the β-amyloid precursor protein (APP) through sequential proteolytic cleavages; first by β-secretase and then by γ-secretase. β-cleavage of APP generates a soluble APPβ (sAPPβ) fragment and a membrane-associated APP β-carboxyl terminal fragment (βCTF). The latter can then be cleaved by γ-secretase to release Aβ. γ-secretase is a high molecular weight complex that consists of presenilin 1 (PS1) or presenilin 2 (PS2), nicastrin, APH1 and PEN-2. Alternatively, APP can be cleaved by α-secretase within the Aβ sequence to release soluble APPα (sAPPα), which is neuroprotective, precluding Aβ generation [1]. Accumulating evidence has shown that overproduction/accumulation of Aβ peptides in vulnerable brain regions is a primary cause in the pathogenesis of AD [2-4]. Therefore, a dysregulation of the proteins involved in Aβ generation, including the altered intracellular trafficking/subcellular localization of them, may increase Aβ generation and lead to disease pathogenesis.

The type I transmembrane aspartyl protease, β-site APP cleaving enzyme 1 (BACE1), is the putative β-secretase [5-8]. Because its optimal activity requires an acidic environment, BACE1 is mainly localized in the early Golgi, late Golgi/early endosomes, and endosomes. In addition, BACE1 can be found at the cell surface [6,9-12]. Although several proteins have been found to interact with BACE1 and regulate its intracellular trafficking, such as reticulon/Nogo [13-15], Golgi-localized γ-ear-containing ARF-binding (GGA) [16-18] and sorting nexin 6 (SNX6) [19], the detailed mechanism underlying BACE1 trafficking regulation has yet to be fully elucidated.

Sorting nexins (SNXs) are a diverse group of cellular trafficking proteins that are unified by the presence of a phospholipid-binding (PX) motif. The ability of SNXs to bind specific phospholipids, as well as their propensity to form protein-protein complexes, suggests a role for these proteins in membrane trafficking and protein sorting [20-22]. Recently, some SNX members have been found to mediate APP processing/Aβ generation through regulating trafficking of AD associated proteins: SNX17 can interact with APP in the early endosome and downregulation of SNX17 leads to reduced steady-state levels of APP with a concomitant increase in Aβ production [23]. SNX33 can bind to the endocytic GTPase dynamin and overexpression of SNX33 reduces the rate of APP endocytosis in a dynamin-dependent manner, so that APP is subjected to increased α-cleavage in cell surface [24]. Moreover, SNX6 can associate with BACE1 and a reduction of SNX6 results in elevated steady-state levels of BACE1 as well as increased retrograde transport of BACE1 in the endocytic pathway, increasing Aβ generation [19]. However, the functional roles of other SNX proteins, especially their involvement in AD, deserve further scrutiny.

In the present study, we demonstrate that another SNX member, SNX12, can interact with BACE1 and affects its intracellular trafficking, thus mediating β-processing of APP in Aβ production.

## Results

### SNX12 affects the level of secreted Aβ

**Figure 1:**
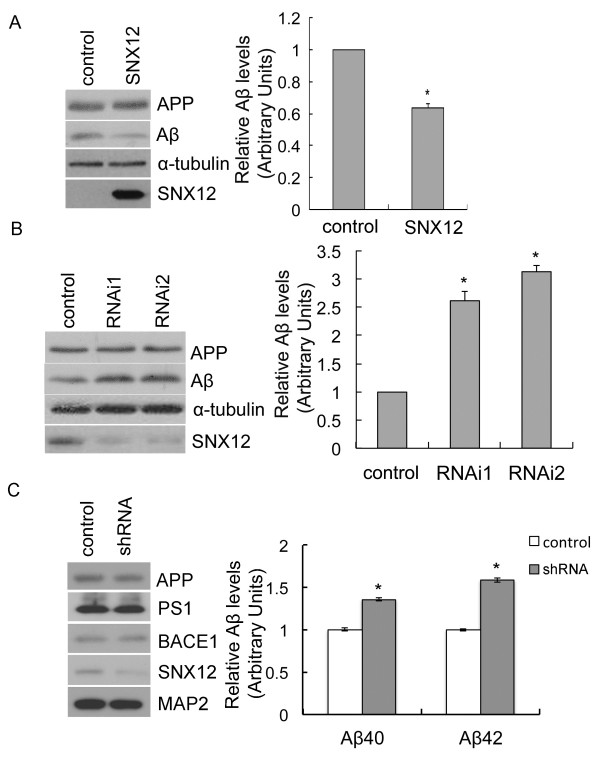
**SNX12 regulates Aβ generation.** HEK-APP_Swe_ cells were transfected with (**A**) SNX12 and control plasmids, or (**B**) two SNX12 siRNAs (1 and 2) and a control scrambled siRNA. Aβ in conditioned media and total APP and SNX12 in cell lysates were analyzed by Western blot. The levels of Aβ were quantified by densitometry for comparison. N = 3, *: *p* < 0.05. (**C**) Primary neurons derived from APP/PS1/tau triple transgenic mice at postnatal day 0 were infected with SNX12 shRNA or control lentiviral particles for 72 h. Neuron lysates were analyzed for APP, PS1-NTF, BACE1, SNX12 and MAP2 by Western blot. Secreted Aβ40 and Aβ42 in conditioned media were quantified by ELISA. The levels of Aβ40 and Aβ42 were normalized to those of controls (set as one arbitrary units) for comparison. N = 3, *: *p* < 0.05.

Several SNX family members have been found to modulate Aβ generation through different mechanisms. Herein, we studied whether several other SNX proteins can also affect Aβ generation. Our results showed that in HEK293 cells stably expressing human APP Swedish mutations (HEK-APP_Swe_), overexpression of one SNX protein, SNX12, dramatically decreased the level of secreted Aβ without affecting the level of full-length APP (Figure 1A). When the level of SNX12 was downregulated by RNAi, the level of full-length APP was not altered but the level of secreted Aβ was significantly increased (Figure 1B). To confirm the regulation of Aβ by SNX12 in primary neurons, we downregulated SNX12 using lentivirus infection in primary neurons derived from APP/PS1/tau triple transgenic mice [25] and found that the levels of Aβ40 and Aβ42 secreted by primary neurons were indeed significantly increased upon downregulation of SNX12 (Figure 1C).

### SNX12 is expressed in the brain and localized in early endosomes

**Figure 2:**
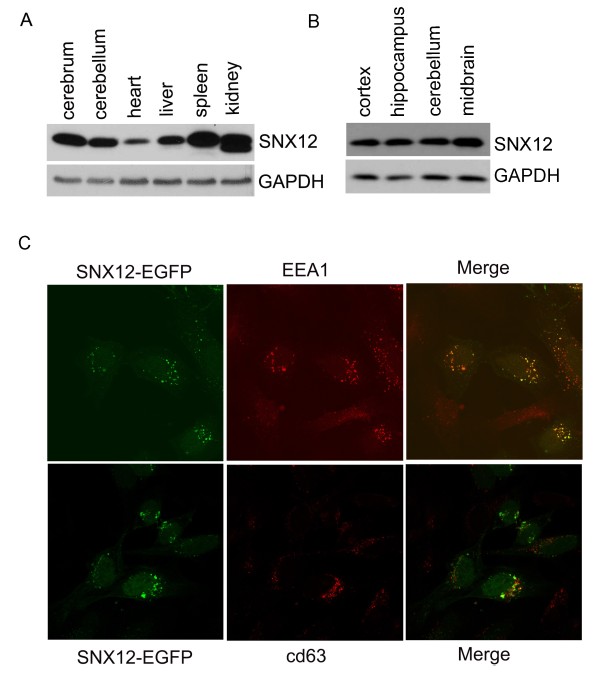
**SNX12 is abundantly expressed in mouse brain tissues and localized in the early endosomes.** The expression of SNX12 in various (**A**) tissues and (**B**) brain regions of two month old C57/Bl6 mice were analyzed by Western blot. (**C**) SNX12-EGFP was transfected into HeLa cells. Cells were fixed, permeabilized, and incubated with antibodies against the early endosome marker EEA1 or the late endosome marker cd63. After additional incubation with Alexa 594-conjugated secondary antibody, cells were examined with a fluorescence confocal microscope. Green: SNX12; red: EEA1 or cd63.

Information on the features and function of SNX12 is very limited. Recently one study reported that SNX12 is widely expressed in the adult mouse central nervous system and thus suggested that SNX12 might regulate neurite formation during cerebral cortical development [26]. Herein, we found that SNX12 is abundantly expressed in mouse cerebrum and cerebellum in addition to other tissues (Figure 2A). Further analysis showed that the expression levels of SNX12 are similar in cortex, hippocampus, cerebellum and midbrain (Figure 2B). Since the major function of the SNX family proteins is thought to be associated with endocytosis, endosomal sorting, endosomal signaling, etc., we speculated that SNX12 may be mainly localized in endosomal compartments. We transfected SNX12-EGFP into HeLa cells and studied the localization of SNX12 in different endosomal compartments by immunofluorescence. The results showed that SNX12 co-localized well with an early endosome marker, EEA1, but not with a late endosome marker, cd63 (Figure 2C), indicating an early endosome localization of SNX12.

### SNX12 does not affect γ-secretase activity

**Figure 3:**
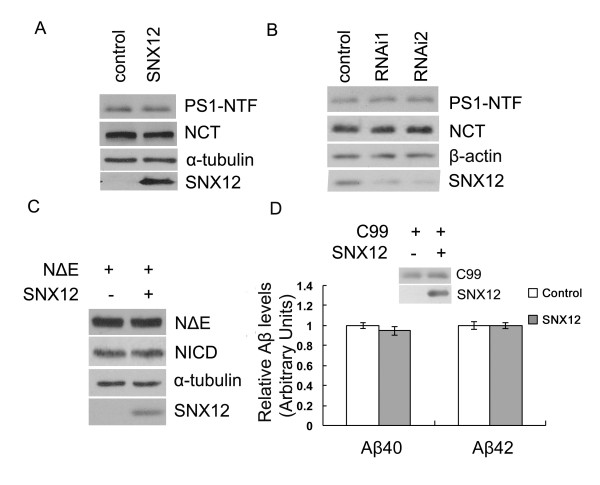
**SNX12 does not affect γ-secretase activity.** HEK-APP_Swe_ cells were transfected with (**A**) SNX12 and control plasmids, or (**B**) two SNX12 siRNAs (1 and 2) and a control scrambled siRNA. Two γ-secretase components, PS1-NTF and nicastrin (NCT), were analyzed by Western blot. (**C**) HEK 293 T cells were first transfected with Notch NΔE. After splitting equally, cells were transfected with SNX12 (+) or control (−) plasmids. Notch NΔE and NICD in cell lysates were analyzed by Western blot. (D) HEK 293 T cells were first transfected with APP βCTF/C99. After splitting equally, cells were transfected with SNX12 (+) or control (−) plasmids. Transfected C99 and SNX12 in cell lysates were analyzed by Western blot. Aβ40 and Aβ42 in conditioned media were assayed by ELISA. N = 4.

To determine how SNX12 affects Aβ, we first investigated whether SNX12 affects γ-secretase. The results showed that neither overexpression (Figure 3A) nor downregulation (Figure 3B) of SNX12 altered the protein levels of presenilin 1 amino-terminal fragment (NTF) and nicastrin (NCT), two major components of γ-secretase. Notch is another major substrate of γ-secretase. When we overexpressed Notch NΔE, which lacks the ectodomain of Notch1 and can be processed in a ligand-independent manner by γ-secretase to produce NICD [27], we found that overexpression of SNX12 did not affect the γ-cleavage of Notch NΔE for NICD production (Figure 3C). In addition, we overexpressed APP βCTF (C99), the direct substrate of γ-secretase, in HEK cells to avoid the interference of β-cleavage, and found that overexpression of SNX12 did not affect the generation of Aβ40 or Aβ42 (Figure 3D). Together, these results suggest that SNX12 does not affect the activity of γ-secretase.

### SNX12 affects the β-processing of APP but not *in vitro* β-secretase activity

**Figure 4:**
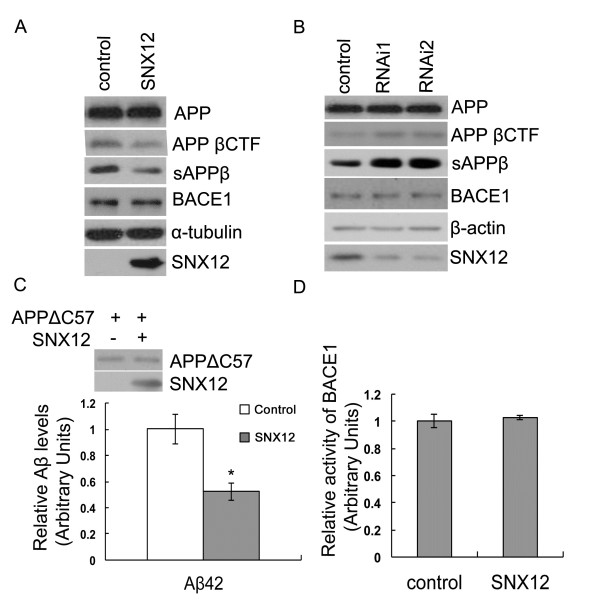
**SNX12 affects β-processing of APP but not BACE1 level or activity.** SH-SY5Y cells were transfected with (**A**) SNX12 and control plasmids, or (**B**) two SNX12 siRNAs (1 and 2) and a control scrambled siRNA. sAPPβ in conditioned media and total APP, APP βCTF and BACE1 in cell lysates were analyzed by Western blot. (**C**) HEK 293 T cells were first transfected with APP ΔC57. After splitting equally, cells were transfected with SNX12 (+) or control (−) plasmids. Transfected APP ΔC57 and SNX12 in cell lysates were analyzed by Western blot. Aβ42 in conditioned media were assayed by ELISA. N = 4, *: *p* < 0.05. (**D**) SH-SY5Y cells were transfected with SNX12 and control plasmids. Cell lysates were assayed for BACE1 activity using a commercial kit. N = 3.

Next, we studied whether SNX12 affects the β-cleavage of APP. Upon overexpression of SNX12, we found that although the total level of BACE1 was not altered, the levels of both APP βCTF and sAPPβ, the two direct products of the β-cleavage of APP, were markedly decreased (Figure 4A). Downregulation of SNX12 increased the levels of APP βCTF and sAPPβ without affecting the level of BACE1 (Figure 4B). APP ΔC57 lacks the last 57 carboxyl-terminal amino acids of APP and cleavage of APP ΔC57 by β-secretase leads to direct production of Aβ42. We found that in cells expressing human APP ΔC57, overexpression of SNX12 significantly reduced the level of secreted human Aβ42 (Figure 4C). Together, these results suggest that SNX12 affects the β-processing of APP. However, when SH-SY5Y cells transfected with SNX12 and control plasmid were compared for *in vitro* activity of BACE1 using a commercial kit, we found no differences between them (Figure 4D), suggesting that SNX12 does not affect the general enzymatic activity of BACE1.

### SNX12 interacts with BACE1 but not APP

**Figure 5:**
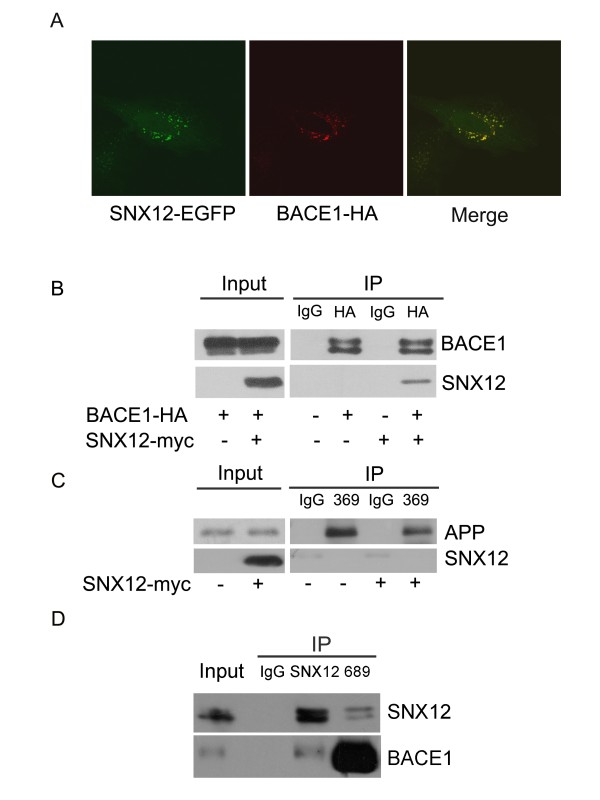
**SNX12 interacts with BACE1.** (**A**) HeLa cells were co-transfected with SNX12-EGFP (in green) and BACE1-HA. Cells were then fixed, permeabilized and immunostained sequentially with primary antibodies against HA and secondary antibodies conjugated with Alexa Fluor 594 (to indicate BACE1, in red). Samples were examined by a fluorescence confocal microscope. (**B**) BACE1-HA was co-transfected with control (−) or SNX12-Myc (+) into HEK 293T cells. Equal protein amounts of cell lysates were used for immunoprecipitation (IP) with an anti-HA antibody or control IgG. Immunoprecipitated proteins were subjected to Western blot with anti-HA or anti-Myc antibodies to detect BACE1 or SNX12, respectively. Ten percent of cell lysates were used as input. (**C**) HEK-APP_Swe_ cells were transfected with control (−) or SNX12-Myc (+). Equal protein amounts of cell lysates were subjected to IP with control IgG or the anti-APP antibody 369. Immunoprecipitated proteins and input were subjected to Western blot. (**D**) Equal protein amounts of SH-SY5Y cell lysates were subjected to IP with control IgG, anti-SNX12 antibody, or the anti-BACE1 antibody 689. Immunoprecipitated proteins and input were subjected to Western blot analysis.

Since SNX12 does not affect the level/activity of BACE1, SNX12 may modulate the β-cleavage of APP through different mechanisms. Because we found that SNX12 is localized in early endosomes and BACE1 has been reported to also be localized in early endosomes, we co-transfected SNX12-EGFP and BACE1-HA into HeLa cells and carried out immunofluorescence studies. The results showed that SNX12 co-localized with BACE1 (Figure 5A). In addition, in cells overexpressing SNX12-Myc and BACE1-HA, an anti-HA antibody pulled down SNX12, indicating an interaction between SNX12 and BACE1 (Figure 5B). However, in cells overexpressing SNX12-Myc and APP, an anti-APP antibody (369) could not pull down SNX12, indicating that SNX12 does not interact with APP (Figure 5C). Moreover, we carried out co-immunoprecipitation between endogenous SNX12 and endogenous BACE1 in SH-SY5Y cells and found that an anti-SNX12 antibody pulled down BACE1 and an anti-BACE1 antibody (689) pulled down SNX12, confirming the interaction between the two (Figure 5D).

### SNX12 regulates the endocytosis of BACE1

**Figure 6:**
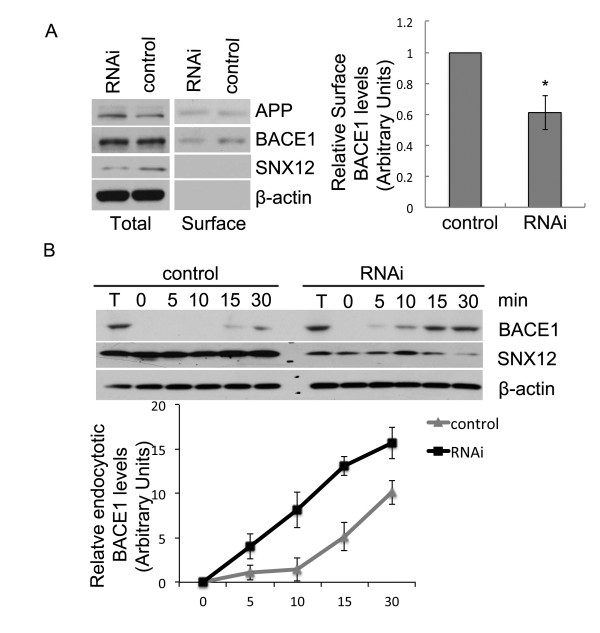
**Downregulation of SNX12 accelerates the endocytosis of BACE1 and decreases the cell surface level of BACE1.** (**A**) After RNAi downregulation of SNX12, SH-SY5Y cells were subjected to biotinylation. Cell lysates were affinity-precipitated with streptavidin-agarose beads to pull down biotinylated proteins that were at the cell surface. The levels of biotinylated BACE1 and APP, as well as their total protein levels, were analyzed by Western blot. Cell surface levels of BACE1 were quantified by densitometry and normalized to that of control (set as one arbitrary unit) for comparison. N = 3, *: *p* < 0.05. (**B**) Upon downregulation of SNX12 by RNAi, protein at the cell surface of SH-SY5Y cells was labeled with EZ-Link Sulfo-SS-Biotin. After chasing at 37 °C for the indicated times, cells were treated with glutathione at 4 °C to cleave biotin from remaining cell surface proteins. Cell lysates were affinity-precipitated with streptavidin-agarose beads. The levels of biotinylated (i.e. endocytosed) BACE1 were analyzed by Western blot. The levels of endocytotic BACE1 were quantified by densitometry and normalized to those of five percent of total (T) BACE1 (before chasing) for comparison. N = 3, *: *p* < 0.05.

Since the endosomal compartment is a major site for the BACE1 cleavage of APP and Aβ generation, SNX12 might mediate the endocytosis of BACE1 through their interaction and thus affect the β-processing of APP. To address this, we carried out a cell surface protein biotinylation assay and found that upon downregulation of SNX12, the level of cell surface BACE1 was markedly decreased, whereas the levels of cell surface APP, total APP and total BACE1 were not affected (Figure 6A). Moreover, we studied endocytosis of cell surface BACE1. Under same conditions, an observable endocytosis of BACE1 in control cells occurred at about 15 min after chasing, whereas in cells with SNX12 downregulation, an observable endocytosis of BACE1 occurred early at 5 min after chasing (Figure 6B), suggesting that downregulation of SNX12 can accelerate the endocytosis of BACE1.

### The level of SNX12 is decreased in the brain of AD patients

**Figure 7:**
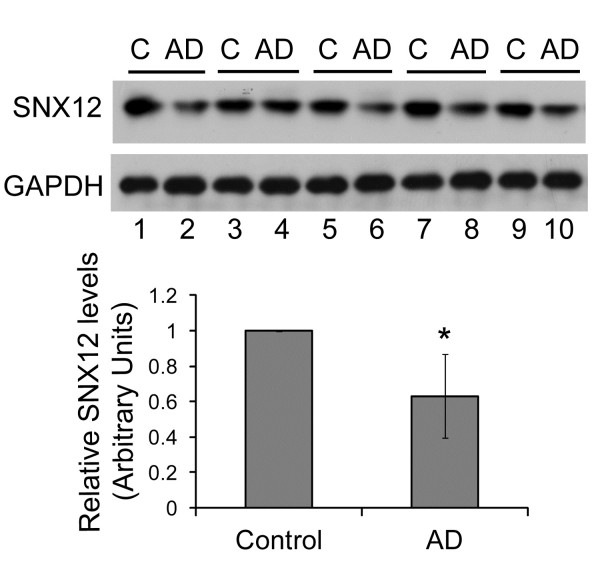
**The level of SNX12 is decreased in AD brains.** Equal amounts of protein lysates from the brains of AD patients and age/sex matched controls (C) were analyzed for SNX12 by Western blot. Protein levels were quantitated by densitometry and normalized to those of GAPDH for statistical comparison to those of controls. Lane numbers are the same as numbers in Table 1. N = 5, *: *p* < 0.05.

Finally, we detected the level of SNX12 in the brain of AD patients and age/sex matched controls. The results showed that the level of SNX12 was dramatically decreased in the brains of AD patients over that in controls (Figure 7), suggesting a direct link between SNX12 changes and AD pathology.

## Discussion

Aβ is generated from its precursor APP through sequential cleavages first by β-secretase/BACE1 and then by γ-secretase, during their highly regulated trafficking within the cell. Dysregulation in the intracellular trafficking of these proteins may alter a proper interaction between APP and its two secretases, thereby affecting Aβ production and contributing to disease pathogenesis. The putative β-secretase, BACE1, cycles between the secretory pathway compartments while BACE1 activity resides in both the secretory pathway and the endosomes. The intracellular trafficking of BACE1 may be affected by molecules that interact with it: some studies found that BACE1 can interact with reticulon/Nogo proteins, whose increased expression can block BACE1 in the ER that has a neutral pH environment and thus inhibit BACE1 activity in Aβ generation [13-15]. In addition, Golgi-localized γ-ear-containing ARF-binding (GGA) proteins interact with BACE1 and regulate its trafficking between the late Golgi and early endosomes; depletion of GGA proteins increases the accumulation of BACE1 in the acidic early endosome for enhanced BACE1 stability and cleavage of APP [16-18]. More recently, a SNX family member, SNX6, has also been found to be associated with BACE1 and a reduction of SNX6 results in elevated steady-state levels of BACE1 as well as increased retrograde transport of BACE1 in the endocytic pathway, causing increased Aβ generation [19].

In this study, we demonstrate that SNX12, another SNX family member, also interacts with BACE1 and affects the β-cleavage of APP. Although the steady-state level of BACE1 was not affected upon modulation of SNX12, downregulation of SNX12 resulted in increased BACE1 endocytosis and Aβ generation, similar to that of SNX6; and overexpression of SNX12 reduced the production of Aβ. Importantly, we found that SNX12 was abundantly expressed in brain tissues and the level of SNX12 was dramatically decreased in the brain of AD patients when compared to that of controls, suggesting a direct link between SNX12 alteration and AD pathology.

SNX family members share a conserved PX motif that acts as a phosphoinositide-binding site for targeting the SNX protein to phosphoinositide-enriched membranes [28]. These proteins have been proposed to play a role in membrane trafficking and protein sorting [20-22]. In addition to SNX12 and SNX6 which interact with BACE1 and affect APP processing/Aβ generation, several other SNX members such as SNX17 [23] and SNX33 [24] have also been found to mediate APP processing/Aβ generation through regulating the intracellular trafficking of APP. Together, these results suggest that SNX members may participate in AD pathology through modulating the trafficking of various AD-associated proteins, which deserves further investigation.

## Conclusion

Our studies have demonstrated that SNX12 may participate in AD pathology by interacting with BACE1, thereby regulating BACE1 intracellular trafficking and BACE1-mediated β-processing of APP in Aβ production. The reduction of SNX12 in the brain of AD patients may accelerate the endocytosis of BACE1 and lead to increased Aβ generation. As BACE1 represents a favored target for AD intervention, inhibition of BACE1-mediated β-processing of APP by regulating SNX12 might provide an alternative strategy for developing AD therapeutics.

## Methods

### Cell culture, plasmids and transfection

HEK293T and SH-SY5Y cells were maintained in DMEM (Hyclone) supplemented with 10% FBS (Hyclone) and 1% penicillin/streptomycin. HEK cells stably expressing human APP Swedish mutations (HEK-APP_Swe_) were maintained in the same media with the addition of 400 μg/mL G418 (Invitrogen). Hela cells were maintained in RPMI-1640 (Hyclone) supplemented with 10% FBS and 1% penicillin/streptomycin. Maintenance of N2a cells stably expressing human APP695 (N2a-APP) was as previously reported [29]. Primary neurons derived from APP/PS1/tau triple transgenic mice collected at postnatal day 0 were cultured in Neurobasal medium supplemented with B-27 (Invitrogen).

The pCI-neo-SNX12/myc plasmid and the pCI-neo control plasmid were kindly provided by Dr. Wanjin Hong. The BACE1-HA plasmid was kindly provided by Dr. Riqiang Yan. The APP βCTF (containing the last 99 carboxyl-amino acids, C99) and ΔC57 (lacking the last 57 carboxyl-terminal amino acids) fragments were inserted into the pcDNA3.1-Myc/His plasmid (Invitrogen) to generate plasmids. The SNX12-EGFP plasmid was constructed by subcloning SNX12 into the pEGFP-N1 plasmid (Clontech). Notch NΔE plasmid was as previously reported [27,30].

Transient transfection was performed by using TurboFect reagent (Fermentas), following the manufacturer’s protocol.

### Antibodies and western blot

Antibodies used in this study include: anti-Myc antibody (9E10) from Santa Cruz Biotechnology; anti-HA and anti-SNX12 antibodies from Gene Tex; anti-α-tubulin and anti-β-actin antibodies and control IgG from Sigma; anti-Aβ antibody (6E10) from Covance; and anti-EEA1 and anti-cd63 antibodies from Abcam. Anti-BACE1 antibody 689 and anti-APP antibody 369 were kindly provided by Dr. Huaxi Xu. Anti-BACE1 antibody 3D5 was kindly provided by Dr. Robert Vassar.

Cells were lysed in a lysis buffer containing 10 mM Tris–HCl (pH 7.8), 1 mM EDTA, 150 mM NaCl, 1% Nonidet P-40 and supplemented with a protease inhibitor mixture. Equal protein amounts of cell lysates were subjected to SDS-PAGE and Western blot with indicated antibodies.

### SNX12 RNA interference

For RNA interference (RNAi) to downregulate human SNX12 expression, two SNX12 targeting siRNAs (1: 5’- ATGAGCTGGAGAGAGATAG-3’; and 2: 5’- TGAACGCTGCCTACACATG-3’) and a scrambled control siRNA (Invitrogen) were transfected into cells using Lipofectamine RNAiMAX reagent (Invitrogen), following the manufacturer’s protocol.

### Lentivirus infection

SNX12 shRNA and control lentiviral particles were purchased from Santa Cruz Biotechnology. These viruses were used to infect APP/PS1/tau primary neurons for 72 h before experiments.

### Immunoprecipitation

Cells were lysed in a lysis buffer. Cell lysates were pre-cleared with rProtein A sepharose (GE) at 4 °C for 1 h and then incubated with rProtein A sepharose and the indicated antibodies or IgG at 4 °C overnight. Immunoprecipitated proteins were analyzed by Western blot.

### Aβ assay

To detect the total level of secreted Aβ, cells were transfected for 24-48 h and then incubated with DMEM for 4-6 h. Conditioned media was then collected and incubated with trichloroacetic acid (1:9 v/v) overnight at 4 °C for protein precipitation. Precipitated proteins were subjected to Western blot with the anti-Aβ antibody 6E10. Alternatively, Conditioned media were assayed by ELISA to detect Aβ40 and Aβ42, using commercial ELISA kits (Invitrogen).

### BACE1 activity assay

The *in vitro* BACE1 activity was assayed by using the β-Secretase Activity Assay Kit, Fluorogenic (Calbiochem), following the manufacturer’s protocol.

### Immunofluorescence

Treated cells were fixed in 4% paraformaldehyde and permeabilized with 0.2% Triton-X100. Cells were then incubated with the indicated primary antibodies, followed by incubation with Alexa 594-conjugated secondary antibody (Invitrogen). Specimens were examined with a ZEISS LSM 5 Exciter confocal microscope and images were captured using the ZEN software.

### Cell surface biotinylation

Treated cells were washed with ice-cold phosphate-buffered saline containing 1 mM each of CaCl_2_ and MgCl_2_, and incubated at 4 °C with 0.5 mg/mL Sulfo-NHS-LC-biotin (Thermo Scientific, Rockford, IL). Cells were then lysed and lysates were affinity-precipitated with streptavidin-agarose beads (Thermo Scientific). Biotinylated proteins were subjected to Western blot analysis.

### Cell surface protein endocytosis assay

Cells were first biotinylated with cleavable EZ-Link Sulfo-SS-Biotin (Thermo Scientific). After chasing at 37 °C for the indicated times, cells were treated with cold glutathione buffer at 4 °C to cleave biotin from cell surface proteins. The remaining biotinylated proteins represent the endocytosed proteins and were affinity-precipitated with streptavidin-agarose beads for further Western blot analysis.

### SNX12 level in human brains

Brain cortical region samples of AD patients and age- and sex-matched controls were kindly provided by Dr. Yong Shen. More detailed information of these samples was listed in Table 1. Samples were lysed in RIPA buffer and equal protein amounts of samples were subjected to Western blot to detect the level of SNX12.

**Table 1:** Human brain samples used in this study

Number*	**Case**	**Sex**	**Age**
1	Non-AD	Female	82
2	AD	Female	83
3	Non-AD	Female	76
4	AD	Female	78
5	Non-AD	Male	83
6	AD	Male	83
7	Non-AD	Male	68
8	AD	Male	67
9	Non-AD	Male	78
10	AD	Male	76

*: Numbers are the same as lane numbers in Figure 7.

### Statistical analysis

Two-tailed paired *t*-test was carried out for statistical analysis.

## Abbreviations

Aβ, β-amyloid; APP, β-amyloid precursor protein; BACE1, β-site APP cleaving enzyme 1; AD, Alzheimer’s disease; CTF, Carboxyl terminal fragment; sAPPβ, NCT Nicastrin; NTF, amino-terminal fragment; PS1, Presenilin 1; PS2, Presenilin 2; RNAi, RNA interference; APPβ, Soluble; SNX, Sorting nexin; SNX12, Sorting nexin 12.

## Competing interests

The authors declare that they have no competing interests.

## Authors’ contributions

YZ, YW, JY, XW and YZ performed experiments; XZ analyzed data; Y-wZ designed research, analyzed data and wrote the paper. All authors have read and approved the final manuscript.
